# The Influence of Conditions of Polycondensation in Acid Medium on the Structure of Oligosilsesquioxanes with a Novel Eugenol-Containing Substituent

**DOI:** 10.3390/polym16202951

**Published:** 2024-10-21

**Authors:** Alexander D. Ageenkov, Nikolay S. Bredov, Anna A. Shcherbina, Ramil R. Khasbiullin, Anton S. Tupikov, Mikhail A. Soldatov

**Affiliations:** 1 Department of Chemical Technology of Polymer Composite Paints and Coatings, Mendeleev University of Chemical Technology, Miusskaya sq. 9, 125047 Moscow, Russia; ageenkov.a.d@muctr.ru; 2 Laboratory of Organoelement Oligomers and Polymers, Mendeleev University of Chemical Technology, Miusskaya sq. 9, 125047 Moscow, Russia; bredov.n.s@muctr.ru (N.S.B.); tupikov.a.s@muctr.ru (A.S.T.); 3 Department of Chemical Technology of Plastic Materials, Mendeleev University of Chemical Technology, Miusskaya sq. 9, 125047 Moscow, Russia; 4 Department of Plastic Processing Technology, Mendeleev University of Chemical Technology, Miusskaya sq. 9, 125047 Moscow, Russia; sherbina.a.a@muctr.ru; 5 Laboratory of Structural and Morphological Research, A.N. Frumkin Institute of Physical Chemistry and Electrochemistry of Russian Academy of Science, Leninsky Pr. 31-4, 119071 Moscow, Russia; khasbiullin@techno-poisk.ru

**Keywords:** siloxane, oligoorganosilsesquioxane, eugenol, polycondensation, active medium

## Abstract

Eugenol-containing oligoorganosilsesquioxanes were synthesized by the method of hydrolytic polycondensation in an active medium under various reaction conditions. The obtained products were characterized by ^29^Si NMR spectroscopy and MALDI-TOF spectrometry. It was shown that factors such as the reaction temperature, polycondensation duration, and molar ratio between the initial alkoxysilane monomer and acetic acid may affect the molecular weight characteristics and molecular structure of the formed oligomer, like the content of stressed cyclic units (T_3_, DTT, TDT) and unstressed silsesquioxane units T_n_D_m_. In particular, an increase in the ratio of the initial reagents led to an increase in the content of silsesquioxane T_n_ fragments from 28.2%_mol_ to 41.7%_mol_, while the number of strained cyclic structures decreased by more than two times. An increase in the synthesis time is of no particular practical value since it was found that the composition of the oligomers synthesized for 6 h and 12 h was practically identical, as was that of the oligomers synthesized for 24 h and 48 h. A noticeable transition in the oligomer composition was observed only when the synthesis time was changed from 12 h to 24 h. Finally, it was shown that the choice of synthesis temperature had the strongest effect on the oligomer composition. The oligomer synthesized at 95 °C contained the highest amount of silsesquioxane T_n_ fragments, >77%_mol_, while a T_n_ fragment content of ~42%_mol_ was observed during the synthesis at 117 °C. It was shown that silsesquioxanes are devitrified at room temperature (T_g_ from −6.4 to −10.6 °C), and their thermal stability in an inert atmosphere is 300 °C. The synthesized oligomers, due to the presence of hydroxyl-containing eugenol units, may be promising binders and additives for functional epoxy–silicone paints and coating materials.

## 1. Introduction

Siloxane-based oligomers have found a wide range of applications in the field of paints and coating materials [1,2,3,4,5,6,7], polymeric composite materials [3,8,9,10,11], etc. There are several approaches to synthesizing such oligomers, and the method of the polycondensation of alkoxysilanes in an active medium has become more popular due to its high efficiency and environmental friendliness [12,13,14]. In this method, anhydrous organic acid, usually acetic acid, is used and plays the role of a solvent, catalyst, and reactant simultaneously. Water, necessary for the hydrolysis of alkoxysilanes, is formed in situ during the reaction process, which allows for fine-tuned reaction conditions and the structure of the formed oligomer as well. Mostly, factors such as reaction temperature [15,16], the ratio between reagents [17], the nature of the substituent on silicon atoms [18,19], and the nature of the formed low-molecular-weight compounds [15,20] can have an influence on the structure of the formed organosilicon oligomers. These factors can have an effect on the mechanism of the formation of siloxane bonds: homogeneous (≡Si–OH + OH–Si≡) or heterogeneous (≡Si–OH + AlkO–Si≡) polycondensation [21,22]. Many various substituents were studied, such as non-functional methyl [23,24] and phenyl [24,25] groups and functional vinyl [13,26], methacrylic [27,28], aminopropyl [29,30], p-aminophenyl [31,32,33], maleimide [34], glycidyl [35,36], and mercaptopropyl groups [12,37]. So, the findings of the novel functional substituent on silicon atoms seem to be promising for the preparation of novel functional organosilicon polymeric materials. In some of their works, Muzafarov et al. have shown that the use of click reactions, like thiol-ene reactions, might be useful for the preparation of novel functional compounds [38,39,40]. Moreover, this type of reaction may be an alternative to the hydrosilylation reaction or Piers–Rubinsztajn reaction [41,42,43], traditionally used in organosilicon chemistry [44]. A key advantage of this reaction consists of its high selectivity, reaction rate, and yield of product in comparison with other reactions [45,46]. This reaction is also technologically simple and safe and enables the use of a large number of organic precursors containing –SH and C=C groups. Also, UV and thermal radical initiators can be used. All these aforementioned factors make the thiol-ene reaction highly prospective for the preparation of novel functional materials.

In their work, Rissing and Son [47] studied the functionalization of tetravinylsilane with –SH-containing compounds, which also contained functional groups such as –OH, –COOH, –NH_2_, and –SO_3_Na. Photoinitiators were used, and the yields of products varied from 64 to 100% depending on the initial precursor. It should be noted that only 1–5% of the product isomers were from Markovnikov substitution. The authors claimed that the obtained compounds may be used for hyperbranched and dendrimer siloxane compounds. A similar reaction was used for octavinylsilsesquioxane in [48]. In [49], thermal initiation with AIBN was studied for the same compounds. It should be noted that in both cases, the yield of the product was almost quantitative. In another study [50], the authors functionalized mercaptopropyltrialkoxysilanes with various vinyl- and allyl-containing compounds. It was reported that the formed compounds may be used for surface grafting onto magnetic nanoparticles of iron oxide.

Therefore, the thiol-ene reaction is a promising tool for the synthesis of organosilicon compounds and can be considered as an alternative to the use of traditional hydrosilylation or Pierce–Rubinstein reactions.

As we noted earlier, the introduction of novel substituents on the silicon atom or secondary functionalization of the silicon atom is a promising task, the solution of which will expand the possibilities of using siloxanes and their precursors as building blocks for obtaining functional materials.

It is well known that a substituent on the silicon atom has a significant effect on the structure of siloxanes or silsesquioxanes and the functional properties of materials based on them.

To date, there are known cases of the modification of synthesized silsesquioxanes with eugenol through polymer-analogous transformations. In one study [51], the modification of the cage-type silsesquioxane T_8_(SH)_8_ with eugenyl methacrylate by polymer-analogous transformation was demonstrated. According to the authors, the modified silsesquioxane obtained better solubility in various solvents and was also a carrier of antibacterial properties. Another study [52] showed a similar modification with 4-maleimidophenol, which has antioxidant properties, resulting in improved antioxidant properties. In the study [53], silsesquioxane T_8_(SH)_8_ was functionalized with a thermosetting phenol–formaldehyde resin, resulting in increased thermal stability during high-temperature oxidation due to the formation of crystalline SiO_2_ during carbonization.

However, modifications of linear and cyclic hydride-containing siloxanes with eugenol are also known, as a result of which the anticorrosive properties of coatings based on them [54,55,56], the dielectric constant [57], or the viscoelastic modulus of gels [58] is increased.

Previously, we synthesized eugenol-containing oligoorganosilsesquioxane by means of hydrolytic polycondensation and polycondensation in an active medium and showed its potential as an anticorrosive coating material [59]. We showed that the choice of the synthetic route significantly influences the properties of obtained oligomers. In this work, we have studied the influence of reaction conditions on the structure of the obtained oligomers for polycondensation in an active medium in further depth.

## 2. Materials and Methods

### 2.1. Materials

Unless otherwise noted, all chemicals were purchased from commercial suppliers and used as received.

S-[(p-hydroxy-m-methoxy)phenylpropyl]-mercaptopropyltrimethoxysilane (EugSSi) was synthesized according to a method given in the Supplementary Materials.

Acetic acid (AcOH) was purchased from the company Russkiy Khimik LLC (Moscow, Russia) and dried according to the standard method by prolonged refluxing with P_2_O_5_ followed by distillation over zeolite 4A [60].

### 2.2. General Procedure for the Synthesis of Eugenol-Containing Oligosilsesquioxanes (OESSs)

EugSSi and AcOH were injected into a round-bottom flask equipped with a reflux condenser. The synthesis was carried out with continuous stirring under heating for a certain time. At the end of the synthesis, the reaction mixture was dissolved in chloroform and washed with NaHCO_3_ 10% solution and then distilled water until the reaction was neutral. After washing, the oligosilsesquioxane solution was dried over MgSO_4_. It was then filtered through a ceramic Schott filter, and the solvents were removed on a rotary evaporator at 50 °C and 725 mmHg pressure. The synthesized oligosilsesquioxane was a brownish-yellow, viscous product.

#### 2.2.1. Synthesis of OESS_1.5

First, 0.1 mol (36.054 g) EugSSi and 0.45 mol (27.02 g) AcOH were injected into a round-bottom flask equipped with a reflux condenser. The synthesis was carried out with continuous stirring at a temperature of 117 °C for 48 h. ^29^Si NMR (400 MHz, Chloroform-d), δ (ppm): −48.58, −53.03, −54.05, −54.81, −55.50, −57.76, −58.50, −59.36, −59.98, −60.86, −63.79, −65.15, −67.51. The yield was 71% by weight.

#### 2.2.2. Synthesis of OESS_2.0

First, 0.1 mol (36.054 g) EugSSi and 0.6 mol (36.03 g) AcOH were injected into a round-bottom flask equipped with a reflux condenser. The synthesis was carried out with continuous stirring at a temperature of 117 °C for 48 h. ^29^Si NMR (400 MHz, Chloroform-d), δ (ppm): −53.57, −54.24, −55.27, −56.23, −57.88, −58.71, −59.62, −60.35, −61.21, −64.20, −67.66. The yield was 67% by weight.

#### 2.2.3. Synthesis of OESS_3.0

First, 0.1 mol (36.054 g) EugSSi and 0.9 mol (54.05 g) AcOH were injected into a round-bottom flask equipped with a reflux condenser. The synthesis was carried out with continuous stirring at a temperature of 117 °C for 48 h. ^29^Si NMR (400 MHz, Chloroform-d), δ (ppm): −53.67, −54.24, −55.28, −56.39, −58.73, −60.44, −65.91, −66.47, −67.72. The yield was 72% by weight.

#### 2.2.4. Synthesis of OESS_6

First, 0.1 mol (36.054 g) EugSSi and 0.9 mol (54.05 g) AcOH were injected into a round-bottom flask equipped with a reflux condenser. The synthesis was carried out with continuous stirring at a temperature of 117 °C for 6 h. ^29^Si NMR (400 MHz, Chloroform-d), δ (ppm): −48.59, −50.78, −54.42, −55.29, −56.96, −58.75, −64.38, −67.83. The yield was 75% by weight.

#### 2.2.5. Synthesis of OESS_12

First, 0.1 mol (36.054 g) EugSSi and 0.9 mol (54.05 g) AcOH were injected into a round-bottom flask equipped with a reflux condenser. The synthesis was carried out with continuous stirring at a temperature of 117 °C for 12 h. ^29^Si NMR (400 MHz, Chloroform-d), δ (ppm): −48.63, −50.77, −54.42, −55.25, −58.78, −67.53. The yield was 69% by weight.

#### 2.2.6. Synthesis of OESS_24

First, 0.1 mol (36.054 g) EugSSi and 0.9 mol (54.05 g) AcOH were injected into a round-bottom flask equipped with a reflux condenser. The synthesis was carried out with continuous stirring at a temperature of 117 °C for 24 h. ^29^Si NMR (400 MHz, Chloroform-d), δ (ppm): −53.04, −54.06, −54.90, −55.55, −57.82, −58.41, −60.00, −60.91, −63.82, −65.17, −66.15, −68.05. The yield was 70% by weight.

#### 2.2.7. Synthesis of OESS_95

First, 0.1 mol (36.054 g) EugSSi and 0.9 mol (54.05 g) AcOH were injected into a round-bottom flask equipped with a reflux condenser. The synthesis was carried out with continuous stirring at a temperature of 95 °C for 48 h. ^29^Si NMR (400 MHz, Chloroform-d), δ (ppm): −55.79, −58.30, −63.95, −64.62, −65.34, −66.38, −67.45, −67.74, −68.15. The yield was 83% by weight.

## 3. Results

**Scheme 1:**
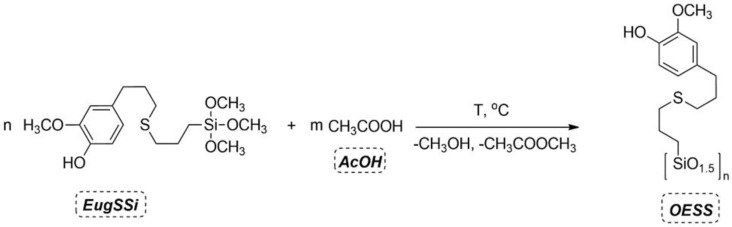
Polycondensation reaction of EugSSi in acid medium.

The OESSs were synthesized by the polycondensation of a eugenol-containing alkoxysilane monomer in a medium of acetic acid according to Scheme 1.

**Scheme 2:**
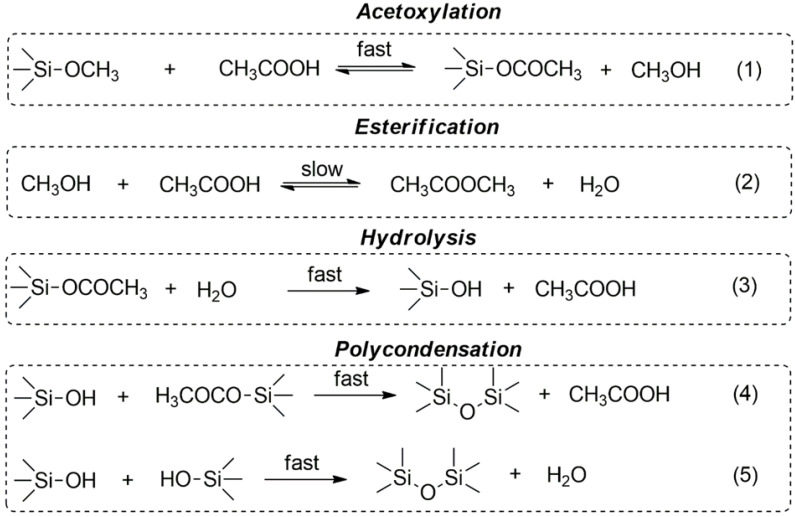
Mechanism of polycondensation of alkoxysilanes in active medium.

It is well known that the polycondensation in an acid medium, also known as acidohydrolytic polycondensation (AHPC), of alkoxysilanes is a series of reversible sequential–parallel stages [61] (Scheme 2). The formation of the siloxane bonds occurs as a result of homo- or heterofunctional condensation between two silanol groups (≡Si–OH + HO–Si≡) or silanol ≡Si–OH and an acetoxysilyl group CH_3_CO–Si≡ (reactions 4 and 5, respectively). The formation of the silanol group occurs as a result of the hydrolysis of acetoxysilyl groups by water (reaction 3), which in turn is formed as a result of the slowest stage of the esterification of acetic acid with the corresponding alcohol (reaction 2).

As mentioned before, AHPC is of considerable interest due to its simplicity and the possibility of fine-tuning the structure of the formed oligomers, as it is carried out in homogeneous conditions in comparison with traditional hydrolytic polycondensation (HPC) of organoalkoxysilanes. In our work, this tuning was performed by varying the reaction conditions, shown in Table 1: the ratio of initial regents EugSSi/AcOH (products OESS_1.5, OESS_2.0, and OESS_3.0), reaction time (products OESS_3.0, OESS_6, OESS_12, and OESS_24), reaction temperature (products OESS_3.0 and OESS_95).

**Figure 1:**
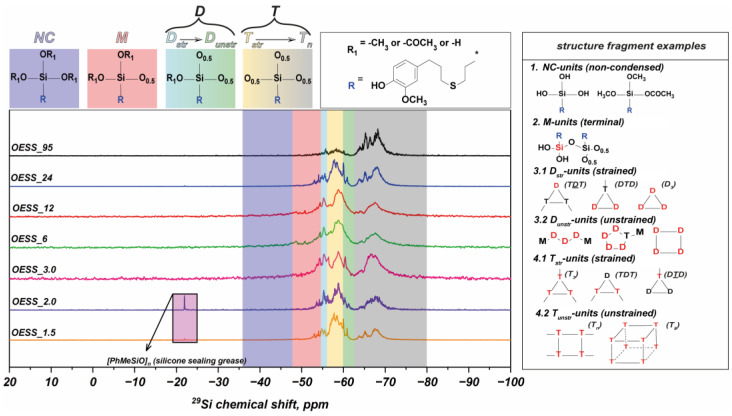
^29^Si NMR spectra of synthesized oligosilsesquioxanes.

On the ^29^Si NMR spectra of the synthesized oligosilsesquioxanes (Figure 1), we can identify six main signal zones. These zones are designated as follows: the NC range (−36.0 to −48.0 ppm), the M range (−48.0 to −54.5 ppm), the D_str_ range (−54.5 to −56.5 ppm), the T_str_ range (−56.5 to −59.5 ppm), the D_unstr_ range (−59.5 to −62.5 ppm), and the T_n_ range (−62.5 to −80.0 ppm). The NC range is characterized by signals from uncondensed Si atoms in various environments. At the initial stages of the transformation, we can find initial monomers, acetoxylation products, or hydrolysis products in this range. The M zone is characterized by structural fragments with a single Si-O-Si bond, which results in these atoms being terminal in the siloxane skeleton. The signals from Si atoms that have undergone acetoxylation appear in a weaker magnetic field compared with hydrolyzed Si-OH atoms, which have a stronger characteristic field. With further condensation, a second siloxane bridge is formed, causing the signals to shift to a higher field—the D_str_ range. In this range, the signals correspond to the stressed Si atoms. It is logical to assume that these signals should be followed by those from unstressed Si atoms. However, in practice, these signals overlap signals from Si atoms in T units, which can be found in stressed cyclic structures such as TTT (T_3_), DTT, and DTD (T_str_ range). After this, there are signals from unstressed Si atoms with two siloxane bonds (D_unstr_ range). Subsequent condensation leads to the formation of a third Si-O-Si bond. The signals from Si atoms with three siloxane bridges appear in the T_n_ region (at n ≥ 4) and correspond to nodal Si atoms, which form the basis of the siloxane backbone. These Si atoms typically correspond to fully condensed ladder-like structures or «closed-cell» types of structures.

### 3.1. Effect of EugSSi/AcOH Molar Ratio

**Figure 2:**
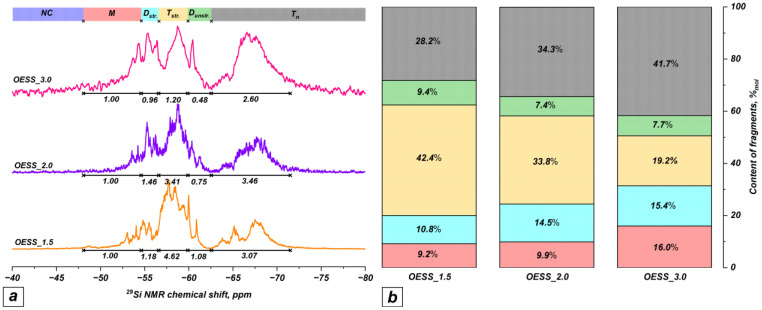
^29^Si NMR spectra (**a**) and histogram structural unit content (**b**) of synthesized oligosilsesquioxanes with various molar ratios of EugSSi/AcOH.

^29^Si NMR spectra for products OESS_1.5, OESS_2.0, and OESS_3.0 with different ratios of EugSSi/AcOH are shown in Figure 2a. The spectra indicate the absence of NC signals for all the oligosilsesquioxane products, which shows that the EugSSi monomer has reacted completely. At −48.6 ppm in this case, the M range is characteristic only for the sample with equimolar ratios of EugSSi/AcOH = 1:1.5 (OESS_1.5), apparently referring to ≡SiOCOCH_3_. Two signals in the range from −53.0 to −54.5 ppm in all spectra correspond to the terminal fragments of the M structure with -OH groups. Two intensive signals from −54.5 to −56.5 ppm correspond to Si atoms in the structures with strained D fragments, most likely having the form of DDD or DDT. The signals in the range from −56.5 to −59.5 ppm corresponding to completely condensed Si atoms of strained T_3_ fragments of the structure are quite intense.

**Figure 3:**
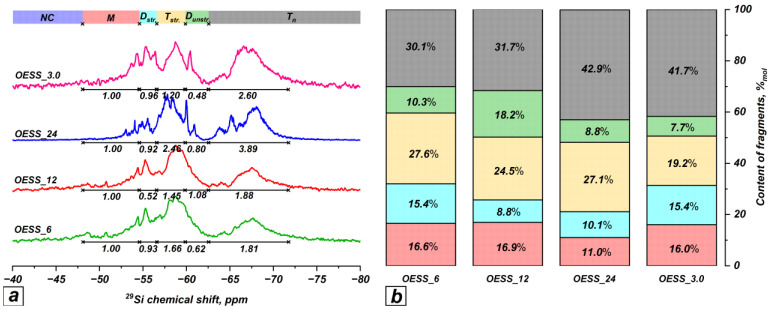
^29^Si NMR spectra (**a**) and histogram structural unit content (**b**) of synthesized oligosilsesquioxanes with various reaction times.

For the OESS_1.5 sample, three main signals are observed in T_str_. Apparently, this is due to the DTD, DTT, and TTT structures. For the OESS_2.0 and OESS_3.0 samples, in this case, two and one signals are present, respectively, which can also be transformed into the signals of Si atoms in the strained DTT and DTD structures. In particular, from −59.5 to −62.5 ppm, signals of Si atoms in unstrained D fragments of the structure are observed, apparently related to cyclic [RSiR_1_O]_n_ structures with n ≥ 4 [62] or to linear fragments. In T_n_, characteristic broad Si signals are observed for all OESS samples, which provide T fragments in ladder-like structures, as well as fragments of the cellular T_8_ type or higher molecular weight [63,64]. From the analysis of the molar amounts of structural fragments in the OESS_1.5, OESS_2.0, and OESS_3.0 samples, one can clearly see an increase in the M, D_str_, and T_n_ fragment amounts and a decrease in the T_str_ fragment amount with an increase in the EugSSi/AcOH ratio (Figure 2b). This is obviously due to the predominance of heterofunctional polycondensation at EugSSi/CH_3_COOH molar ratios of 1:1.5 and 1:2, characterized by a higher process rate compared to homofunctional. As a result, the content of M units is low (does not exceed 10%) due to the resulting Si–OH groups with a high probability for immediate reaction with functional Si–OCH_3_ or Si–O(O)CCH_3_ groups. In addition, for the same reason, the molar content of T_str_ units has higher values than that at a EugSSi/AcOH ratio of 1:3. It is likely that strained structures are formed at the initial stages of the polycondensation process, when the interacting molecules contain a significant number of functional Si–OCH_3_ and Si–O(O)CCH_3_ groups. At a EugSSi/CH_3_COOH ratio of 1:3, a higher amount of silanol groups is present in the reaction system. Due to the increase in acetic acid content, the dilution of the reaction mixture takes place, leading to a decrease in the oligomer concentration, which, as a result, leads to a longer time for the polycondensation process to form fully condensed structures, which, as can be seen from the results of this study (Figure 3), are predominantly unstrained.

### 3.2. Effect of Reaction Time

An increase in the synthesis time under conditions of excess AcOH suggests an increase in the degree of condensation and the formation of more spatially complex structures. The study of the structures of oligosilsesquioxanes was carried out at four time points of EugSSi polycondensation—6, 12, 24, and 48 h. ^29^Si NMR spectra for products obtained after various reaction times are presented in Figure 3a. Here one can see the full absence of NC signals again, which indicates full conversion of the initial monomer. For samples OESS_6 and OESS_12, signals are observed at −48.6 and −50.8 ppm, respectively, which are not typical for samples OESS_24 and OESS_3.0. These signals probably correspond to Si atoms, containing unreacted −OCH_3_ and −OCOCH_3_ groups formed due to the acetoxylation process.

At the same time, the total amounts of M units are comparable (Figure 3b), which means a high content of silanol end-groups for the samples OESS_24 and OESS_3.0 compared to samples OESS_6 and OESS_12. The contents of D_str_ and T_str_ fragments are apparently statistical in nature and do not depend on the synthesis time. In this case, the number of unstrained T_n_ fragments of the structures increases by approximately 1.4 times with the increase in synthesis time, which may be associated with further intermolecular condensation of ≡Si-OH groups in unstrained D_unstr_ fragments. This can be indirectly confirmed by a decrease in the molar concentration of D_unstr_ fragments. On the other hand, it can be concluded that after 24 h of reaction, the content of T_n_ units almost does not change, which may indicate that the equilibria state of siloxane bond formation has been achieved, and there is no need to increase the reaction time after 24 h, which may be an important factor from a technological point of view.

### 3.3. Effect of Reaction Temperature

**Figure 4:**
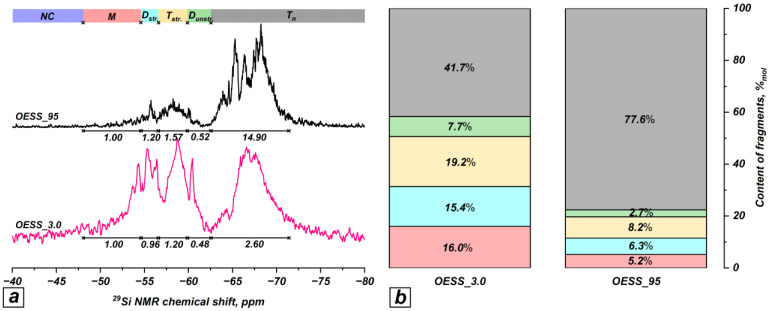
^29^Si NMR spectra (**a**) and histogram structural unit content (**b**) of synthesized oligosilsesquioxanes with various reaction temperatures.

It is assumed that the change in temperature affects the rate of individual stages of the oligosilsesquioxane polycondensation reaction. The ^29^Si NMR spectra of OESSs obtained at 95 °C and 117 °C are shown in Figure 4a. As one can see, no clear intense Si signals can be observed in the M range for OESS_95, which may apparently be due to a higher degree of condensation and a low content of M units of about 5.2% (Figure 4b). A similar behavior is observed for the D_st_., T_str_, and D_unstr_ ranges, where the molar content of fragments does not exceed 10%. In this case, the signals of Si atoms in the T_n_ fragments for the product obtained at 95 °C are almost two times higher than the content of similar T_n_ fragments for the sample obtained at 117 °C. A very important difference in the synthesis temperatures is that the methanol, formed during the acetoxylation of methoxysilyl groups and acetic acid, apparently evaporates from the system at a lower rate, which accordingly affects its concentration in the system and the rate of the esterification reaction with acetic acid. This, in turn, affects the rate of water formation and its concentration.

In addition, a lower temperature promotes a lower evaporation rate of water from the system. In connection with this, it is likely that the relatively high concentration of water in the system leads to rapid hydrolysis of acetoxylated Si atoms and rapid further heterofunctional polycondensation. Moreover, the higher temperature may lead to partial cleavage of siloxane bonds, which in turn increases the content of silanol groups and M units.

### 3.4. Study of Molecular Weight Characteristics

**Figure 5:**
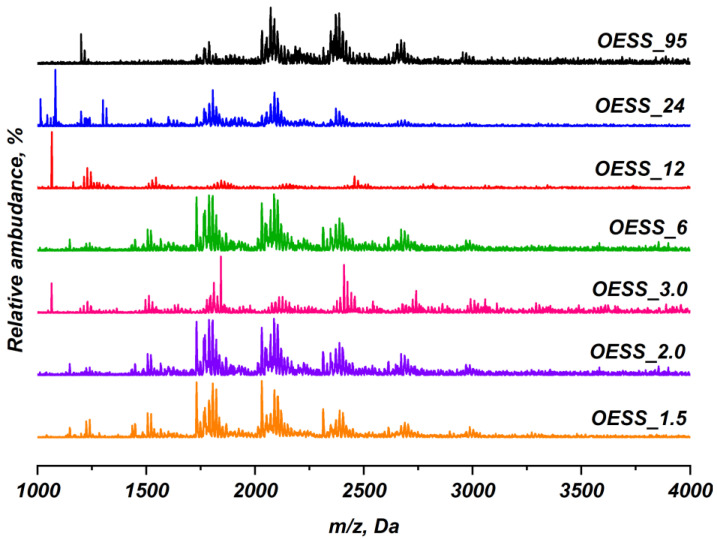
MALDI-TOF mass spectra of the obtained products.

An analysis of the MALDI-TOF mass spectra (Figure 5) of the OESSs shows a weak correlation with the data on the structure of oligosilsesquioxanes determined by ^29^Si NMR spectroscopy. The mass spectra of OESSs, obtained at different molar ratios of EugSSi/AcOH, have similar behavior. They mainly contain *m*/*z* signals of cyclosiloxanes D_4_–D_8_ in various ionic forms with H^+^, Li^+^, Na^+^, and K^+^, as well as an insignificant content of compounds with *m*/*z* corresponding to the structures with silsesquioxane units, for example, T_4_D_2_ (*m*/*z* = 1767), T_2_D_5_ (*m*/*z* = 2085), etc. It is obvious that the bulk of silsesquioxane oligomers is not subject to ionization due to the specific nature and bulkiness of organic substituents on silicon atoms. Thus, the observed pattern reflects the molecular weight characteristics of only a small fraction of the oligomeric compounds present in OESSs that were ionized and detected. Therefore, the use of MALDI-TOF mass spectrometry for a comprehensive study of the structure and molecular weight characteristics of OESSs is not quite appropriate and demands a deeper study.

**Figure 6:**
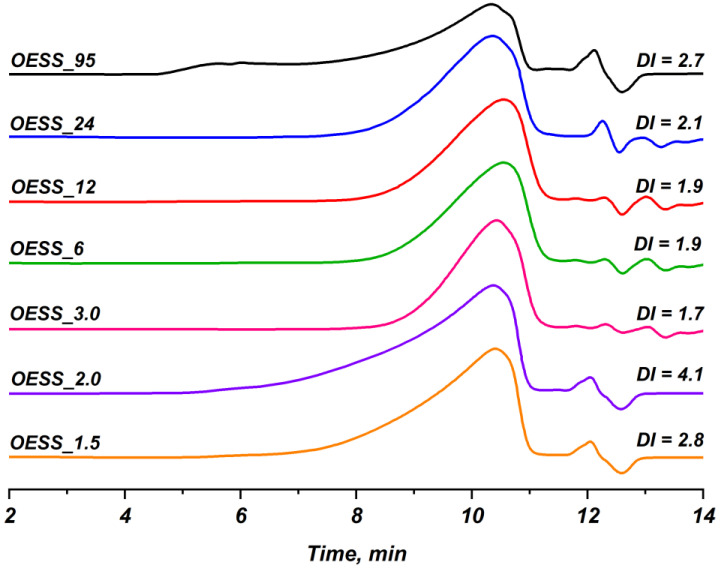
GPC curves of the obtained products.

Molecular weight characteristics were also studied by gel-permeation chromatography (GPC, Figure 6). In a comparison of GPC chromatograms, it was found that in all experiments, the relative molecular weight is similar and is characterized by the molecular weight of the main peak M_n_ ~ 3650–3850. The most indicative characteristic turned out to be the dispersity index DI (Table 2). When the EugSSi/AcOH ratio was varied, it turned out that the sample OESS_3.0, synthesized in an excess of acetic acid, had the narrowest DI, DI = 1.7. We assume that this effect is caused by the possible solvation of molecules during synthesis. In contrast, samples OESS_1.5 and OESS_2.0, synthesized at the ratios EugSSi/AcOH = 1:1.5 and 1:2, have a DI more than 1.5 times higher (2.9 and 4.1, respectively). Thus, it was shown that the narrowest DI of oligosilsesquioxanes with varying ratios of initial reagents can be formed at EugSSi/AcOH = 1:3.

When studying the polycondensation time of OESS samples, we also found that there was no significant effect on the molecular weight M_n_. As the synthesis time increased, only DI decreased from ~2 at 6, 12, and 24 h to 1.7 at 48 h.

The most interesting sample was OESS_95, obtained at 95 °C. It shows two distinct fractions. The first higher-molecular-weight step corresponds to the onset of the oligomer release time after 3.5 min with a peak retention time of 4.6 min and PDI = 1.1. The main fraction of the oligosilsesquioxane has an onset of the release time of 5.9 min with a peak retention time of 9.3 min and DI = 2.7. Apparently, this effect is due to the high content of T_n_ fragments in the structure (>77% according to ^29^Si NMR).

### 3.5. Study of Temperature Transitions and Thermogravimetry

It is known that T_g_ depends on the mobility of the polymer chains, which in turn depends on the substituent and the crosslinking degree. Previously [59], we showed the absence of any thermal transitions in the range from −80 to 250 °C except for the T_g_ for silsesquioxanes obtained by the AHPC and HPC methods. We have shown that silsesquioxanes with the new eugenol-containing substituent cannot be cured by deeper polycondensation due to the length and bulkiness of this substituent.

**Figure 7:**
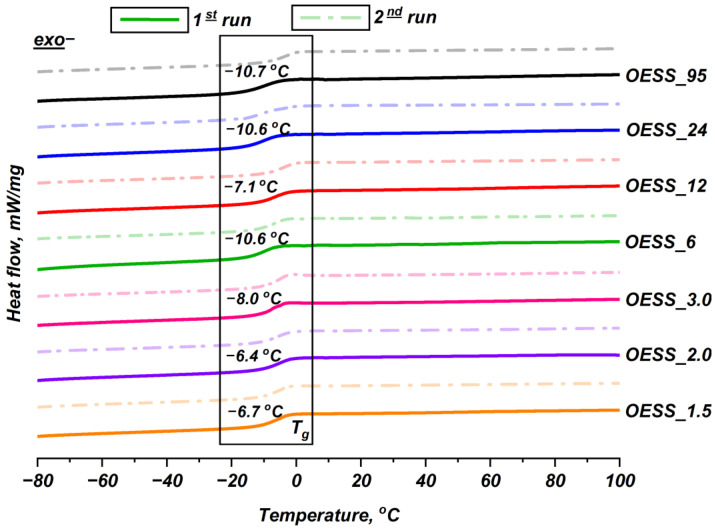
DSC curves of the silsesquioxanes.

In this case, the T_g_ values of all synthesized silsesquioxanes are in the range from −6.4 to −10.7 °C (Figure 7). This means that the silsesquioxanes are in a devitrified state at room temperature.

We attribute this to the presence of the flexible mobile sulfide spacer. At the same time, the difference between the T_g_ values is also due to the composition of the oligomer mixture. Table 3 shows data from DSC and the crosslinking densities (1/M_c_) of silsesquioxanes calculated on the basis of them.

It can be seen that the difference in T_g_ of silsesquioxanes is insignificant. The second run of heating leads to a slight regular increase in T_g_, which may be due to the condensation of individual fragments of the molecule to deeper degrees of transformation. Using the Nilsson formula, we calculated the crosslink density from the difference in T_g_ of the two heatings (without taking into account the chain rigidity of silsesquioxanes). A comparison of crosslinking density is valid only within one group of polycondensation conditions. Thus, within each group, the crosslinking density correlates with the content of silsesquioxane T_n_ links. In this case, there is no correlation between the T_g_ and the content of T_n_ fragments of silsesquioxanes. This is basically true, since the synthesized silsesquioxanes are a complex mixture of linear, cyclic, and self-organized structures.

**Figure 8:**
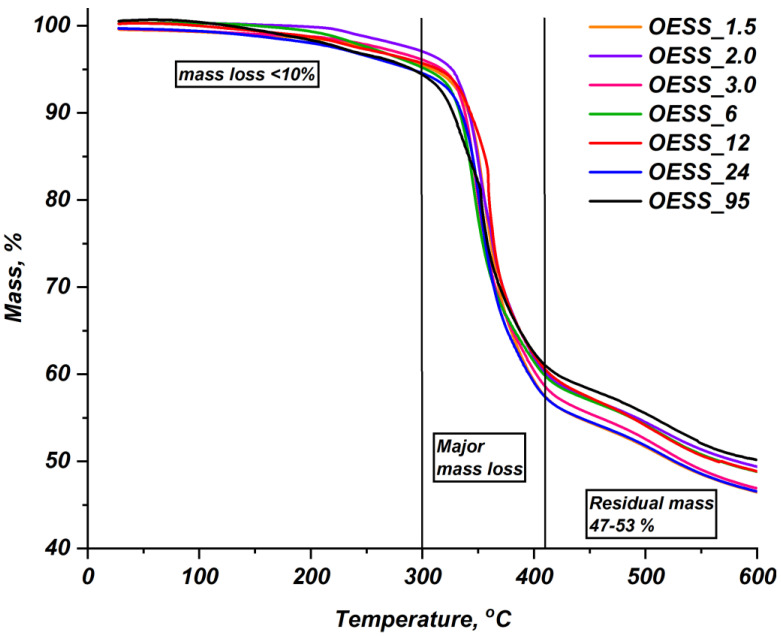
TG curves of the silsesquioxanes.

It is known that silsesquioxanes are quite thermally stable. This stability mainly depends on the organic substituent and the environmental conditions (air or inert atmosphere). To assess thermal stability, we conducted an experiment with heating to 600 °C in an argon atmosphere (Figure 8). The TG curves of silsesquioxanes do not differ significantly from each other. Up to 300 °C, the mass loss is less than 10%. At this stage, polycondensation can occur to deeper degrees, or self-assembly of various oligomeric structures can occur. The main mass loss occurs in the range from 300 to 410 °C. This is primarily due to the rupture of the C-S bond (272 kJ/mol), the C-C bond (346 kJ/mol), and then the Si-O bond (450 kJ/mol). We also assume that in addition to the water and carbon dioxide released during destruction and decomposition, the formation of hydrogen sulfide or sulfur dioxide gas, which are strong corrosive agents, may occur. The mass loss occurs uniformly in one stage. The residual weight at 600 °C is from 47 to 53%. Such a high weight is apparently due to the oxidation and decomposition of silsesquioxanes to SiO_2_.

### 3.6. Effect of Eugenol-Containing Substituent

In the context of the influence of the substituent on the structures of the resulting siloxanes, we are talking more about the predominance of steric factor and hydrophobic interactions [64,65,66]. As a rule, a bulk substituent leads to the formation of products with a low degree of condensation (n). On the other hand, the bulk substituent prevents the gelation process and therefore allows almost completely condensed structures to be obtained [67]. It is known that obtaining a homopolymer mixture based on methylsilsesquioxane is a very labor-intensive task due to its high tendency for gelation [68,69] with the formation of infusible and insoluble products. The most studied systems to date remain phenylsilsesquioxanes [70], which are solid transparent products that can be converted to an infusible and insoluble state when exposed to high temperatures (curing). Their glass transition temperature, as a rule, exceeds 60–200 °C.

In our case, a novel substituent is a molecule consisting of two parts, with methoxyphenol on one side and silyl on the other. The two parts of the molecule are connected to each other by a mobile sulfur-containing molecular spacer (dipropyl sulfide spacer). Regardless of the conditions of the synthesis, the eugenol-containing silsesquioxanes were highly viscous resins devitrified at room temperature. The glass transition temperature of silsesquioxanes is in the low-temperature region (−6.4 ÷ −10.7 °C), which is ensured by the dipropyl sulfide spacer. The novel bulky substituent is supposed to be conducive to predominantly intramolecular interactions during the condensation [66]. At the same time, unlike classical carbon-chain alkyl substituents, its structure contains heteroatoms, which apparently reduce the hydrophobicity of the molecule as a whole, thereby limiting intramolecular condensation and the formation of products with cyclic and self-organized structures. Moreover, as we noted before, the size and volume of the new substituent lead to the formation of oligomeric rather than polymeric products. The molecular weight depends weakly on the polycondensation conditions. At the same time, the new substituent provides moderate heat resistance of the oligomers up to 300 °C.

## 4. Conclusions

A series of oligosilsesquioxanes with a novel eugenol-containing substituent based on S-[(p-hydroxy-m-methoxy)phenylpropyl]-mercaptopropyltrimethoxysilane, which in turn was obtained by the UV-initiated hydrothiolation reaction from commercially available eugenol and 3-mercaptopropyltrimethoxysilane, were synthesized under active-medium conditions. The relationship between the structure of the resulting oligosilsesquioxanes with a novel substituent and the conditions of their synthesis was established for the first time. The combined effect of the new organic substituent and the ratio of the initial reagents, time, and temperature of polycondensation on the structure of the oligosilsesquioxanes was shown. It was found that the greatest amount of T_n_ silsesquioxane structures (>77%_mol_) was formed as a result of polycondensation at 95 °C. By varying the conditions of the polycondensation reaction, the possibility to tune the molecular weight characteristics and dispersity index of novel oligosilsesquioxanes has been demonstrated. The glass transition temperatures of oligosilsesquioxanes were determined to be from −6.4 to −10.6 °C. A correlation was also found between the crosslinking density and the content of T_n_ units within each group of polycondensation conditions. We found that the thermal stability in an inert atmosphere is moderate at temperatures of up to 300 °C, regardless of the reaction conditions. Based on the data about the structure of the elementary repeating units of the oligomers and the structures that have been formed, we suggest the possibility of using these products as adhesives and protective coatings.

## Figures and Tables

**Table 1:** Conditions for the synthesis of oligosilsesquioxanes at a temperature of 117 °C and the content of the resulting structural units.

Product	Molar Ratio of EugSSi/AcOH	Reaction Time, h	Content of Structural Units in Oligoorganosiloxanes, % mol					
			NC	M	D_str._	T_str._	D_unstr._	T_n_
OESS_1.5	1:1.5	48	−	9.2	10.8	42.4	9.4	28.2
OESS_2.0	1:2.0	48	−	9.9	14.5	33.8	7.4	34.3
OESS_3.0	1:3.0	48	−	16.0	15.4	19.2	7.7	41.7
OESS_6	1:3.0	6	−	16.6	15.4	27.6	10.3	30.1
OESS_12	1:3.0	12	−	16.9	8.8	24.5	18.2	31.7
OESS_24	1:3.0	24	−	11.0	10.1	27.1	8.8	42.9
OESS_95 *	1:3.0	48	−	5.2	6.3	8.2	2.7	77.6

* The synthesis was carried out at a temperature of 95 °C.

**Table 2:** Molecular weight characteristics from GPC analysis relative to polystyrene standards.

Product	M_w_	M_n_	DI
OESS_1.5	10,700	3800	2.8
OESS_2.0	15,300	3700	4.1
OESS_3.0	6300	3700	1.7
OESS_6	7000	3600	1.9
OESS_12	7000	3600	1.9
OESS_24	8900	4200	2.1
OESS_95	10,400	3800	2.7

**Table 3:** DSC data and calculating crosslink density.

Product	Glass Transition T_g_ (°C)		Crosslink Density * (1/M_c_)∙10^−2^ (mol/kg)	T_n_-Unit Content According to ^29^Si NMR (%_mol_)
	1st Run	2nd Run		
OESS_1.5	−6.7	−6.5	0.513	28.2
OESS_2.0	−6.4	−6.1	0.749	34.3
OESS_3.0	−8.0	−6.7	3.333	41.7
OESS_6	−10.6	−7.7	7.436	30,1
OESS_12	−7.1	−5.0	5.385	31.7
OESS_24	−10.6	−9.7	2.308	42.9
OESS_95	−10.7	−4.6	15.641	77.6

* M_c_ was calculated using the Nilsson formula (Supplementary Materials).

## Data Availability

Data are contained within the article.

## Supplementary Materials

The following supporting information can be downloaded at https://www.mdpi.com/article/10.3390/polym16202951/s1. Figures S1 and S2, characterization methods, Synthesis of S-[(p-hydroxy-m-methoxy)phenylpropyl]-mercaptopropyltrimethoxysilane (EugSSi).
