# Supplementary material for: The Influence of Conditions of Polycondensation in Acid Medium on the Structure of Oligosilsesquioxanes with a Novel Eugenol-Containing Substituent
Source: Polymers (Basel). 2024 Oct 21;16(20):2951. doi: 10.3390/polym16202951 (PMC11510820; doi:10.3390/polym16202951)
Supplement: Supplementary file 1 [file polymers-16-02951-s001.zip › polymers-3232310-supplementary.pdf]

Supporting Information

The influence of conditions of polycondensation in acid medium on the structure of oligosilsesquioxanes with a novel eugenol-containing substituent

Alexander D. Ageenkov [1,2], Nikolay S. Bredov [2,3], Anna A. Shcherbina [4,5], Ramil R. Khasbiullin [5], Anton S. Tupikov [2,3] and Mikhail A. Soldatov [1,*]

[1] Department of Chemical Technology of Polymer Composite Paints and Coatings, Mendeleev University of Chemical Technology, Miusskaya sq. 9, 125047 Moscow, Russia; ageenkov.a.d@muctr.ru

[2] Laboratory of Organoelement Oligomers and Polymers, Mendeleev University of Chemical Technology, Miusskaya sq. 9, 125047 Moscow, Russia; bredov.n.s@muctr.ru (N.S.B.); tupikov.a.s@muctr.ru (A.S.T.)

[3] Department of Chemical Technology of Plastic Materials, Mendeleev University of Chemical Technology, Mi-usskaya sq. 9, 125047 Moscow, Russia

[4] Department of Plastic Processing Technology, Mendeleev University of Chemical Technology, Miusskaya sq. 9, 125047 Moscow, Russia; sherbina.a.a@muctr.ru

[5] Laboratory of Structural and Morphological Research, A.N. Frumkin Institute of Physical Chemistry and Elec-trochemistry of Russian Academy of Science, Leninsky Pr. 31-4, 119071 Moscow, Russia; khasbiullin@techno-poisk.ru

* Correspondence: soldat89.89@gmail.com

1 Characterization methods

^{1}H, ^{13}C NMR spectra were recorded at a frequency of 600 MHz on a Bruker WB Avance III NMR spectrometer using $CDCl_3$ as a solvent.

^{29}Si NMR spectra were recorded at 400 MHz on a Varian Inova 400 NMR spectrometer with a One QUOTE 400 X/H-F-05 probe using $CDCl_3$ as a solvent. Signal acquisition for ^{29}Si NMR was carried out for 12 hours for each sample.

To record MALDI-TOF mass spectra (MALDI), a Shimadzu Biotech Axima Confidence MALDI tandem mass spectrometer with a nitrogen laser, axial orthogonal irradiation geometry and a recorded power (337 nm) was used. Sample

preparation was carried out in the following order: 8-10 mg of analyzed substances were dissolved in THF, a solution of matrices of 2,5-dihydroxybenzoic acid (DHB) with a concentration of 20 mg/ml in THF was prepared, without the cationizing agent. Next, 20 μL of each analyte solution was mixed with 20 μL of 2,5-dihydroxybenzoic acid matrix solution. 2 μL of each sample was applied to the steel substrate targets until the solvent was completely evaporated.

Fourier transform infrared (FTIR) spectra were recorded on a Jasco FT-IR-4600 spectrometer with an ATR attachment (sapphire tip). The studies were carried out in the wavelength range from 4600 to 300 cm^{-1} with the CO_2 subtraction function.

The study of molecular mass characteristics by gel permeation chromatography (GPC) was carried out on a Shimadzu device with two detectors - RID 20A refractometer, SPD-M20A photodiode matrix, Phenogel 500A column (size (300x7.8 mm)); eluent – tetrahydrofuran; temperature – 40 °C; flow rate 1 ml/min.

DSC thermograms were obtained using Netzsch DSC 204 F1 Phoenix device with a heating rate of 10 K/min in a argon medium. The feed rate of Ar was 50 ml/min. The flow rate of the protective gas was 50 ml/min. TG were obtained using Netzsch TG 209 F1 Iris device with a heating rate of 10 K/min in a argon medium. The feed rate of Ar was 50 ml/min. The crosslinking density was calculated taking into account the difference in glass transition temperatures of two heatings using the Nilsson formula without taking into account the rigidity of the oligosilsesquioxane chain. Nilsson formula (Tg-Tg0) = ke/Mc (ke is a universal polymer constant, equivalent to 39 kg K/mol).

2 Synthesis of S-[(p-hydroxy-m-methoxy)phenylpropyl]-mercaptopropyltrimethoxysilane (EugSSi)

2.1 Characteristics of the initial compounds

4-allyl-2-methoxyphenol (***Eug***) – the product of the company «Dullberg Konzentra» Gmbh, content of the main substance 99.7%, refractive index n_D^{20} =

1.541, ^{1}H NMR (600 MHz, Chloroform-d) δ (ppm): 6.96 (d, 1H), 6.82 – 6.73 (m, 2H), 6.06 (ddt, 1H), 5.89 (d, 1H), 5.23 – 5.11 (m, 2H), 3.90 (s, 3H), 3.41 (dtd, 2H).

3-mercaptopropultrimethoxysilane (***MPTMS***) – the product of the company «JHCHEM» Ltd., content of the main substance 98.0 %, refractive index n_D^{20} = 1.445, ^{1}H NMR (600 MHz, Chloroform-d) δ (ppm): 3.44 (d, 9H), 2.41 (q, 2H), 1.65 – 1.53 (m, 2H), 1.24 (t, 1H), 0.68 – 0.57 (m, 2H).

2-hydroxy-2-methylpropiophenone (***Darocure 1173***) – the product of the company «Suzhou Leba Chemical» Ltd., content of the main substance 97.0 %, refractive index n_D^{20} = 1.533, ^{1}H NMR (600 MHz, Chloroform-d) δ (ppm): 7.99 – 7.94 (d, 2H), 7.57-7.55 (dd, 1H), 7.41 – 7.35 (m, 2H), 4.22 (s, 1H), 1.55-1.38 (d, 6H).

2.2 Synthesis of S-[(p-hydroxy-m-methoxy)phenylpropyl]-mercaptopropyltrimethoxysilane (EugSSi)

In a quartz glass beaker, 0.5 mol (82.35 g) of ***Eug***, 0.5 mol (100.17 g) of ***MPTMS*** and 0.001 mol (0.1693 g) of ***Darocure 1173*** were successively added. The beaker with the mixture was placed on a magnetic stirrer, a UV lamp (395 nm, 600W, 1100mJ/cm2) was turned on, and the synthesis was carried out with continuous stirring for 24 hours at a temperature of 25 °C. The reaction was monitored using a FTIR spectrometer by the disappearance and formation of the corresponding absorption bands. The synthesized S-[(p-hydroxy-m-methoxy)phenylpropyl]-mercaptopropyltrimethoxysilane (***EugSSi***) was a transparent oily liquid of pale yellow color with an odor characteristic of mercaptans and eugenol.

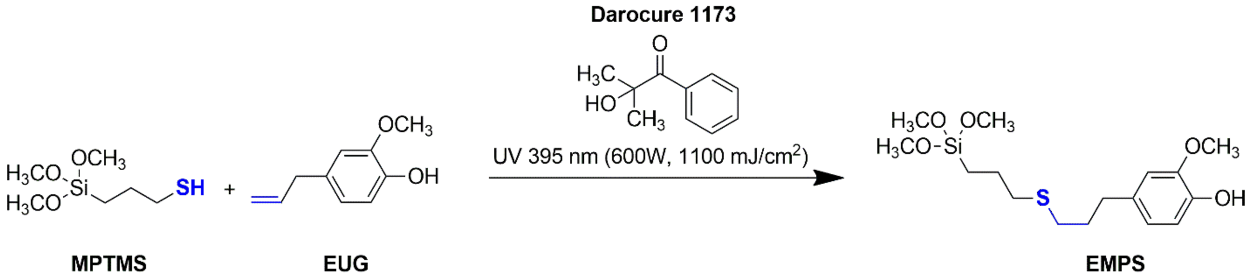

Scheme S1. Synthesis of EugSSi

The reaction was monitored by FTIR spectroscopy based on the decrease in the ν(C=C) band in $H_2C{=}CH{-}CH_2{-}$ at 1638 cm^{-1} (Figure S1). The degree of conversion of ***Eug*** was determined based on the change in the integral intensity of the ν(C=C) band in $H_2C{=}CH{-}CH_2{-}$ at 1638 cm^{-1} using an internal standard – the ν(C−C)$_{ar}$ band in the benzene ring at 1605 cm^{-1} (formula S1).

$$100-(\%CH_2=CH-)=100-\left(\frac{[S_{(C=C)}/S_{(C=C)_{ar}}]_i}{[S_{(C=C)}/S_{(C=C)_{ar}}]_{EUG+MPTMS+Darocure\ 1173}}\cdot 100\right),\quad (S1)$$

где $S_{(C=C)}$ – integrated intensity of the ν(C=C) band in $CH_2{=}CH{-}CH_2{-}$ at 1638 cm^{-1},
$S_{(C-C)ar}$ – integrated intensity of the ν(C−C) in aromatic ring at 1605 cm^{-1}.

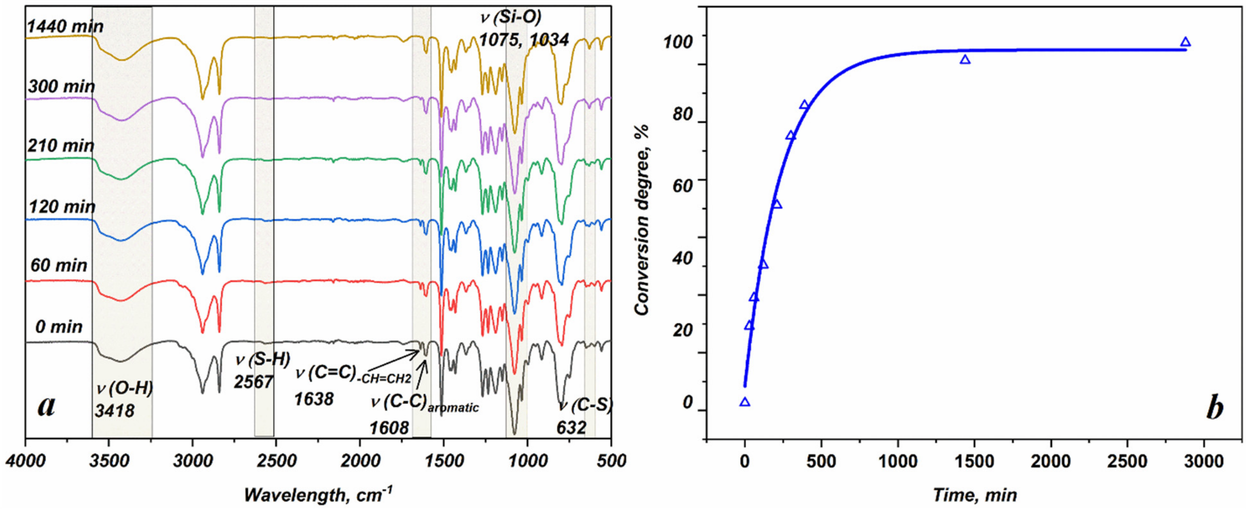

Figure S1 – In line FTIR spectroscopy of ***EugSSi*** formation (a) and conversion degree of ***Eug*** on synthesis time (b)

As can be seen from Figure S1 (b), a conversion degree of more than 70% is achieved already after 5 hours from the start of the reaction, which can be primarily due to the rapid occurrence of the rate-limiting chain transfer stage for thiol-ene reactions [30, 31]. This is also facilitated by the mutual solubility of all reaction components (kinetic region). The conversion yield to the plateau (~ 1000 min) can be due to the low concentration of $-CH_2{-}CH{=}CH_2$ groups (~ 0.0247 mol, 5%) ***Eug*** and −SH in ***MPTMS***.

The most obvious confirmation of the formation of ***EugSSi*** are the ^{1}H and ^{13}C NMR spectra (Figure S2). ***EugSSi*** ^{1}H NMR (600 MHz, Chloroform-d), δ (ppm): 6.78 (d, J = 7.9 Hz, 1H), 6.68 – 6.59 (m, 2H), 5.83 (d, J = 1.4 Hz, 1H), 3.86

– 3.78 (m, 3H), 3.59 – 3.51 (m, 9H), 2.60 (t, J = 7.5 Hz, 2H), 2.48 (dt, J = 11.1, 7.3 Hz, 4H), 1.83 (p, J = 7.3 Hz, 2H), 1.75 – 1.58 (m, 2H), 0.79 – 0.67 (m, 2H). ***EugSSi*** ^{13}C NMR (600 MHz, Chloroform-d), δ (ppm): 146.39, 143.69, 133.32, 120.82, 114.25, 110.98, 55.67, 50.42, 34.86, 34.34, 31.38, 31.06, 2 2.88, 8.44.

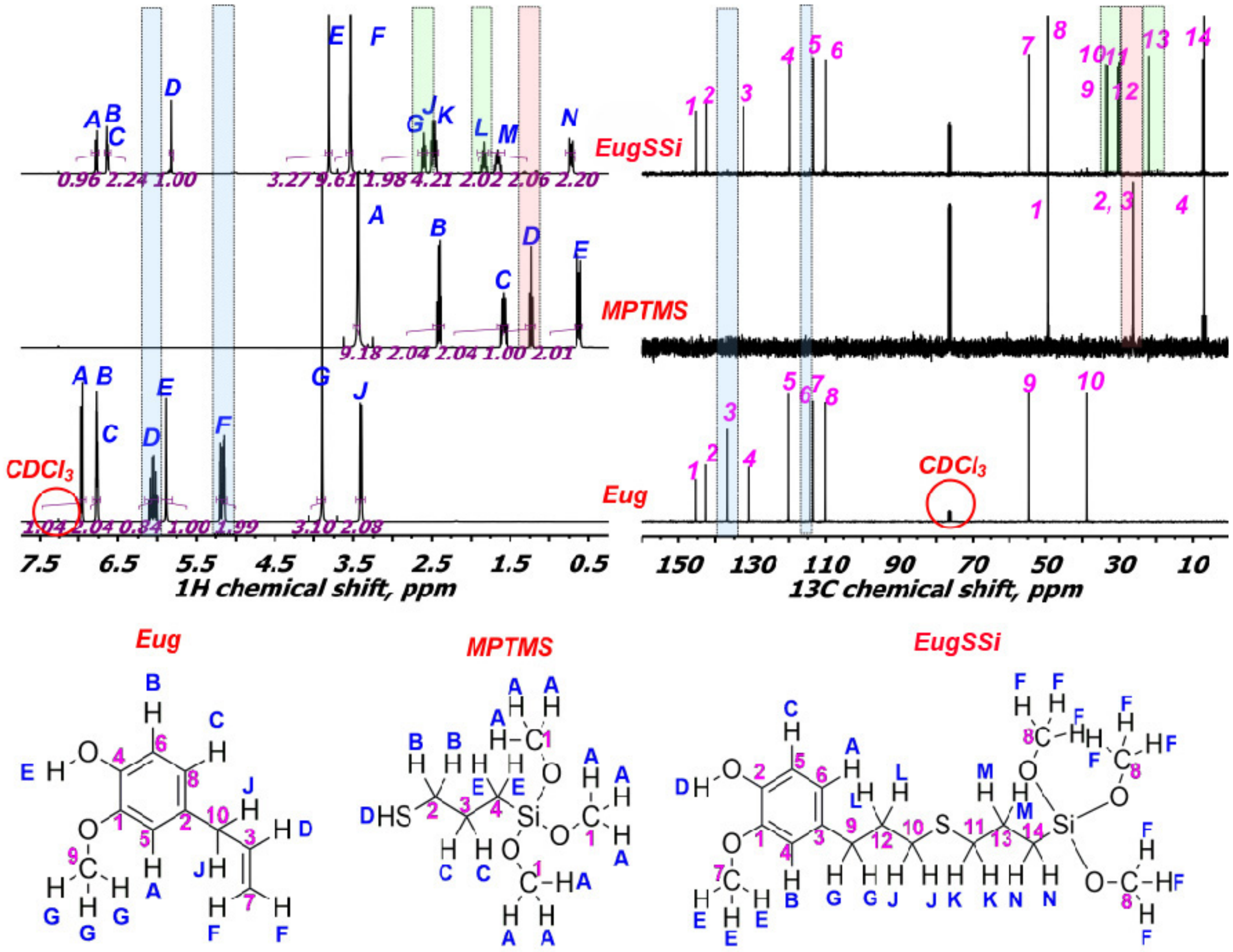

Figure S2 – ^{1}H, ^{13}C NMR spectra of eugenol (***Eug***), 3-mercaptopropyltrimethoxysilane (***MPTMS***) and S-[(p-hydroxy-m-methoxy)phenylpropyl]-mercaptopropyltrimethoxysilane (***EugSSi***)

The ^{1}H NMR spectrum shows the disappearance of signals D (6.06 ppm, 1H) and F (5.23-5.11 ppm, 2H) in ***Eug***, corresponding to the −CH$_2$−CH=C**H$_2$** protons of the allylic group, as well as the disappearance of signal D (1.24 ppm, 1H) in ***MPTMS***, corresponding to the **H**S− proton of the mercapto group. In this case, a covalent bond is formed between the allylic group of ***Eug*** and the mercapto group of ***MPTMS***, as a result of which signals J (2.48 ppm, 2H), K (2.48 ppm, 2H) and L (1.83 ppm, 2H) appear in ***EugSSi***, corresponding to the Ar protons

...−H_2C−H_2C−S−CH_2−...Si. Due to the formation of a new covalent bond, a redistribution of the electron density of atoms occurs, and the signal J (3.41 ppm, 2H) in ***Eug*** shifts to a stronger field − the signal G (2.60 ppm, 2H) in ***EugSSi***. The ratio of the integral intensities of protons corresponds to the calculated one.

Similar patterns of signal changes are observed for the ^{13}C NMR spectrum. The signals of carbon atoms 3 and 7 in ***Eug*** are not observed on a par with atoms 2 and 3 in ***MPTMS***. With the formation of a covalent bond between the allyl group of ***Eug*** and the mercapto group of ***MPTMS***, signals 10 and 11 are formed. However, signals that could correspond to an isomeric product of anti-Markovnikov addition were not detected by us.
